# Association between tea consumption and cognitive impairment in middle-aged and older adults

**DOI:** 10.1186/s12877-020-01848-6

**Published:** 2020-11-04

**Authors:** Jia Zhang, Anxin Wang, Xiaoli Zhang, Shuohua Chen, Shouling Wu, Xingquan Zhao, Qian Zhang

**Affiliations:** 1 Department of Neurology, Beijing Tiantan Hospital, Capital Medical University, No.119, South 4th Ring West Road, Fengtai District, Beijing, 100070 China; 2 China National Clinical Research Center for Neurological Diseases, Beijing Tiantan Hospital, Capital Medical University, Beijing, China; 3 Department of Cardiology, Kailuan Hospital, North China University of Science and Technology, Tangshan, 063000 China

**Keywords:** Cognitive impairment, Tea consumption, Middle-aged adults, Older adults

## Abstract

**Background:**

Biologic studies have suggested that tea may have neuroprotective activity. However, tea’s protective effect on cognitive function is controversial in human epidemiological studies, and no data, including the middle-aged, are available. The objective of this study was to investigate the association of habit, frequency, and types of tea consumption with incident cognitive impairment in middle-aged and older adults.

**Methods:**

Data from the Asymptomatic Polyvascular Abnormalities in Community study were used (aged over 40y). We gathered information on tea consumption, including habit, frequency, and types, via a standardized questionnaire and assessed cognitive function by Mini-Mental State Examination (MMSE) and/or Montreal Cognitive Assessment (MoCA). Three thousand eight hundred sixty-eight and 806 participants were selected in MMSE and MoCA subgroups. Multivariate logistic regression models were utilized to examine associations between tea consumption and cognitive impairment in middle-aged and older participants.

**Results:**

In MMSE analyses, after adjustment for potential confounding factors, habitual (odds ratio (OR) 0.47, [95% confidence interval (CI) 0.33–0.68], *p* < 0.001) and high frequency (p trend < 0.001) of tea intake were associated with a lower risk of cognitive impairment. The risk of cognitive impairment was lower in green tea consumption (OR 0.36, [95% CI 0.22–0.61], *p* < 0.001) than other types (OR 0.59, [95% CI 0.38–0.91], *p* = 0.017). In MoCA analyses, we got similar results.

**Conclusions:**

Habitual tea consumption, especially high-frequency and green tea consumption, was significantly associated with a lower prevalence of cognitive impairment in middle-aged and older individuals.

**Supplementary Information:**

The online version contains supplementary material available at 10.1186/s12877-020-01848-6.

## Background

Dementia is a significant public health issue resulting in the aging of population and requires urgent addressing and prevention [1, 2]. Dementia afflicts about 50 million people and increases rapidly. The number of patients with dementia will be expected to reach 152 million in 2050 [1]. This disease brings immense burdens to the social economy and families. Due to the limitation of treatments available for dementia, the emphasis has been placed on preventing the disease by modifying risk factors [3]. Patients with dementia have pathological changes in the brain during the middle-age, leading to cognitive decline [4, 5]. The cognitive function of the middle-aged need to be examined. Furthermore, identifying risk factors for cognitive impairment in the middle-aged could help avoid and delay the development of dementia.

Tea drinking was originated in China, and now, it has since spread throughout the whole world. Green, oolong, and black tea, as generally consumed forms of tea, originated from the plant *Camellia sinensis* and different in the manufacturing process [6]. Green tea is a popular beverage in Asia, whereas black tea in Western countries [7]. Epidemiologic studies have examined that tea helped prevent cardiovascular disease and mortality [8, 9]. Experimental and animal studies demonstrated that tea polyphenols might have neuroprotective activity and be useful for neurodegenerative disease therapy in the future [10, 11]. In recent years, researchers have suggested that tea consumption is linked to a lower risk of cognitive disorders [12, 13], whereas others held inconsistent results [14, 15]. Notably, the relationship between habit, frequency, or types of tea consumption and cognitive function, including the middle-aged population, has not been investigated. Therefore, this study was designed to clarify the association of habit, frequency, and types of tea consumption with incident cognitive impairment among adults over 40.

## Method

### Study design and population

**Fig. 1:**
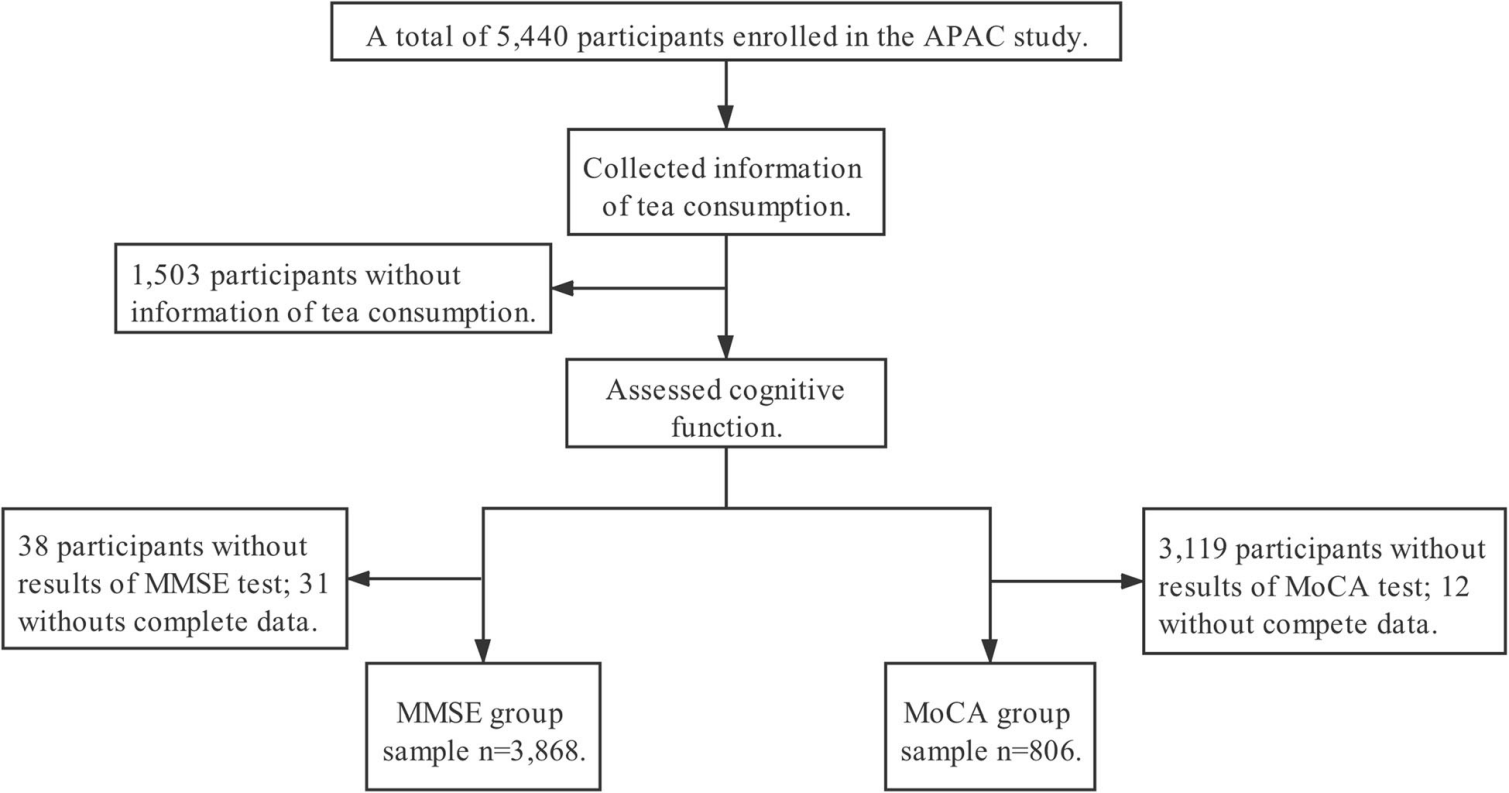
Flow chart of the study

The Asymptomatic Polyvascular Abnormalities in Community (APAC) study is a cross-sectional, community-based, and observational investigation designed to explore the epidemiology of asymptomatic polyvascular abnormalities in Chinese adults. The APAC study population was a part of the previously described Kailuan cohort [16, 17], with 101,510 participants (18–98 years old). In 2010, 7000 participants aged over 40 were randomly selected from the Kailuan study by a random sampling method stratified by age and sex. A total of 5852 people agreed to take part in the APAC study, and eventually, 5816 participants completed the baseline data collection. Three hundred seventy-six participants who did not meet the inclusion criteria were excluded. The inclusion criteria of the APAC study were: (i) over the age of 40; (ii) no history of transient ischemic attack, stroke, or coronary heart disease supplied by participants or their legal representatives at baseline interview; and (iii) absence of neurologic deficits for the previous stroke examined by experienced clinicians. A total of 5440 participants were finally eligible in the APAC study. In this study, information about tea intake and cognitive assessment were collected, and participants were divided into two subgroups for statistical analyses according to cognitive function evaluation methods. We further excluded participants without information of tea consumption (*n* = 1503), results of Mini-Mental State Examination (MMSE) test (*n* = 38) or Montreal Cognitive Assessment (MoCA) (*n* = 3119), and complete data (*n* = 31, for MMSE group; *n* = 12 for MoCA group), leaving 3868 and 806 participants included in MMSE and MoCA subgroups, respectively (Fig. 1). According to the Helsinki Declaration, this study was performed and was approved by the Ethics Committees of Beijing Tiantan Hospital and Kailuan General Hospital.

### Assessment of cognitive function

MMSE and/or MoCA measured cognitive. MMSE is one of the most common and popular cognitive status measurements, but it has a ceiling effect to identify mild cognitive impairment [18]. However, compared to MMSE, MoCA includes more cognition domains tests and presents higher sensitivity and specificity for evaluating early and mild cognitive disorders [19–22]. In this study, to confirm the results, we also used MoCA to detect mild cognitive impairment and performed the analysis. Both the total score of MMSE and MoCA is 30-points [19, 23]. An aggregate score of MoCA needs to be added 1 point when the education level is less than 12 years if the total score is less than 30-points [24]. Cognitive impairment is defined as a score of MMSE < 24 [25], or a score of MoCA < 26 [26].

### Assessment of tea consumption

We collected information about tea consumption via a standardized questionnaire. People were asked to report the habit, the average frequency, and the type of tea they consumed in the past 5 years. The habit is divided into “never” and “habitual.” For the frequency of tea consumption, we provided five choices, including “never,” “less than once/month,” “1–3 times/month,” “1–3 times/week,” and “≥ 4 times/week.” The type of tea consumption included “never,” “green tea,” “black tea,” and “other types” [27].

### Potential covariate assessment

Standardized questionnaires collected demographic information (e.g., age and sex), level of education, lifestyle behaviors (including smoking, alcohol consumption, and physical activity), and medical history (including hypertension, diabetes mellitus, and dyslipidemia). Body mass index (BMI) was calculated as weight (kg) divided by height in meters squared (m2), and classified into three categories according to BMI levels, as “ideal: < 25 kg/m^2^” “intermediate: 25–29 kg/m^2^,” and “poor: ≥ 30 kg/m^2^.” Physical activity was classified by the time of moderate or vigorous activity per week, as “≥ 80 min,” “1–79 min,” and “0 min.” Salt intake was categorized into “< 6 g/day,” “6–10 g/day,” and “> 10 g/day.” [17] Biochemical parameters including plasma high-sensitivity C-reactive protein (hs-CRP), fasting blood glucose, total cholesterol, triglycerides, low-density lipoprotein cholesterol, and high-density lipoprotein cholesterol were measured using an auto-analyzer (Hitachi 747; Hitachi, Tokyo, Japan) at the central laboratory of the Kailuan General Hospital. Hypertension was defined as having a self-report history of hypertension, systolic blood pressure ≥ 140 mmHg, diastolic blood pressure ≥ 90 mmHg, or taking antihypertension agents. Diabetes mellitus was defined as having a self-report history of diabetes mellitus, a fasting blood glucose level ≥ 7.0 mmol/l, or treated with oral hypoglycemic drugs or insulin. Dyslipidemia was defined as the presence of a history of dyslipidemia, triglycerides level ≥ 1.7 mmol/l, total cholesterol ≥5.17 mmol/l/ [28].

### Data analysis

We conducted statistical analyses of different subgroups, respectively, because we classified participants in two subgroups according to cognitive function measurements. Statistical analyses were performed using SAS software (version 9.4; SAS Institute Inc., Cary, NC, USA). Continuous variables were presented as mean ± standard deviation (SD) and compared using ANOVA or Kruskal-Wallis. Categorical variables were presented as frequencies and percentages and compared using the chi-squared test. The types of tea consumption were grouped as “never,” “green tea,” or “others (including black tea and other types).” Logistic regression analysis was used to assess associations between habit, frequency, and types of tea consumption and cognitive function. We fit three multivariate logistic models. The crude model was the unadjusted model. Model 1 was adjusted for age and sex. Model 2 was adjusted for age, sex, and education level. Model 3 was adjusted for all the covariates in model 2 plus smoking, alcohol consumption, hypertension, diabetes mellitus, dyslipidemia, plasma concentrations of hs-CRP, BMI, physical activities, and salt intake. During the regression analysis, we divide the frequency of tea consumption into four groups: never, ≤ 3 times/month, 1–3 times/week, or ≥ 4 times/week. The “never” group of tea consumption was treated as the reference group. Odds ratio (OR) and 95% confidence interval CI) were calculated. All the statistical tests were two-sided, and differences with *p* < 0.05 were regarded as statistically significant.

## Results

The baseline characteristics of participants, according to MMSE assessment, were presented in Table 1. Participants with normal cognition detected by MMSE were most likely to be younger and female, have a higher education level, lower prevalence of hypertension, consume less salt, and prefer tea, especially green tea. In MoCA subgroup analyses, the baseline characteristics were similar (shown in Table 2).

**Table 1:** Baseline characteristics of participants according to MMSE assessment

	MMSE	
	Normal (*n* = 3673)	Cognitive impairment (*n* = 195)
Age, years	56.76 ± 10.54	67.49 ± 12.20†
Sex, n (%)		
Female	1614 (43.94)	59 (30.26) †
Male	2059 (56.06)	136 (69.74)
Education		
Illiteracy or primary school	768 (20.91)	81 (41.54) †
Middle school	1891 (51.48)	104 (53.33)
High school or above	1014 (27.61)	10 (5.13)
Smoking, n (%)	1444 (39.31)	87 (44.62)
Alcohol consumption, n (%)	1122 (30.55)	56 (28.72)
Hypertension, n (%)	1827 (49.74)	131 (67.18) †
Diabetes mellitus, n (%)	490 (13.34)	36 (18.46)
Dyslipidemia, n (%)	1657 (45.11)	73 (37.44) †
Hs-CRP, mg/L	1.95 ± 3.03	2.60 ± 4.42†
BMI		
Ideal	2026 (55.16)	101 (51.79)
Intermediate	1428 (38.88)	81 (41.54)
Poor	219 (5.96)	13 (6.67)
Physical activity		
≥ 80 min	1264 (34.41)	75 (38.46)
1–79 min	1787 (48.65)	92 (47.18)
0 min	622 (16.93)	28 (14.36)
Salt intake		
< 6 g/day	361 (9.83)	24 (12.31) †
6–10 g/day	2158 (58.75)	97 (49.74)
> 10 g/day	1154 (31.42)	74 (37.95)
Tea consumption, n (%)		
Never	2269 (61.78)	150 (76.92) †
Habitual	1404 (38.22)	45 (23.08)
Frequency of tea consumption, n (%)		
Never	2269 (61.78)	150 (76.92) †
Less than once/month	61 (1.66)	0
1–3 times/month	238 (6.48)	8 (4.10)
1–3 times/week	327 (8.90)	8 (4.10)
≥ 4 times/week	778 (21.18)	29 (14.87)
Types of tea consumption, n (%)		
Never	2269 (61.78)	150 (76.92) †
Green tea	735 (20.01)	18 (9.23)
Others	669 (18.21)	27 (13.85)

Data are presented as N, n (%) or mean ± SD

Hs-CRP, plasma high-sensitivity C-reactive protein

†There were significant differences between the no proteinuria group and the proteinuria for one or more times group (*p* < 0.05)

**Table 2:** Baseline characteristics of participants according to MoCA assessment

	MoCA	
	Normal (*n* = 441)	Cognitive impairment (*n* = 365)
Age, years	60.08 ± 12.36	62.05 ± 11.70†
Sex, n (%)		
Female	164 (37.19)	214 (58.63)
Male	277 (62.81)	151 (41.37)
Education		
Illiteracy or primary school	92 (20.86)	96 (26.30) †
Middle school	195 (44.22)	198 (54.25)
High school or above	154 (34.92)	71 (19.45)
Smoking, n (%)	180 (40.82)	154 (42.19)
Alcohol consumption, n (%)	143 (32.43)	101 (27.67)
Hypertension, n (%)	246 (55.78)	224 (61.37)
Diabetes mellitus, n (%)	50 (11.34)	68 (18.63) †
Dyslipidemia, n (%)	174 (39.46)	173 (47.40) †
Hs-CRP, mg/L	2.04 ± 3.02	2.63 ± 4.70†
BMI		
Ideal	254 (57.60)	184 (50.41) †
Intermediate	168 (38.10)	151 (41.37)
Poor	19 (4.31)	30 (8.22)
Physical activity		
≥ 80 min	172 (39.00)	135 (36.99)
1–79 min	160 (36.28)	146 (40.00)
0 min	109 (24.72)	84 (23.01)
Salt intake		
< 6 g/day	38 (8.62)	43 (11.78)
6–10 g/day	244 (55.33)	195 (53.42)
> 10 g/day	159 (36.05)	127 (34.79)
Tea consumption, n (%)		
Never	233 (52.83)	242 (66.30) †
Habitual	208 (47.17)	123 (33.70)
Frequency of tea consumption, n (%)		
Never	233 (52.83)	242 (66.30) †
Less than once/month	8 (1.81)	3 (0.82)
1–3 times/month	38 (8.62)	16 (4.38)
1–3 times/week	41 (9.30)	20 (5.48)
≥ 4 times/week	121 (27.44)	84 (23.01)
Types of tea consumption, n (%)		
Never	233 (52.83)	242 (66.30) †
Green tea	125 (28.34)	51 (13.97)
Others	83 (18.82)	72 (19.73)

Data are presented as N, n (%) or mean ± SD

Hs-CRP, plasma high-sensitivity C-reactive protein

†There were significant differences between the no proteinuria group and the proteinuria for one or more times group (*p* < 0.05)

**Fig. 2:**
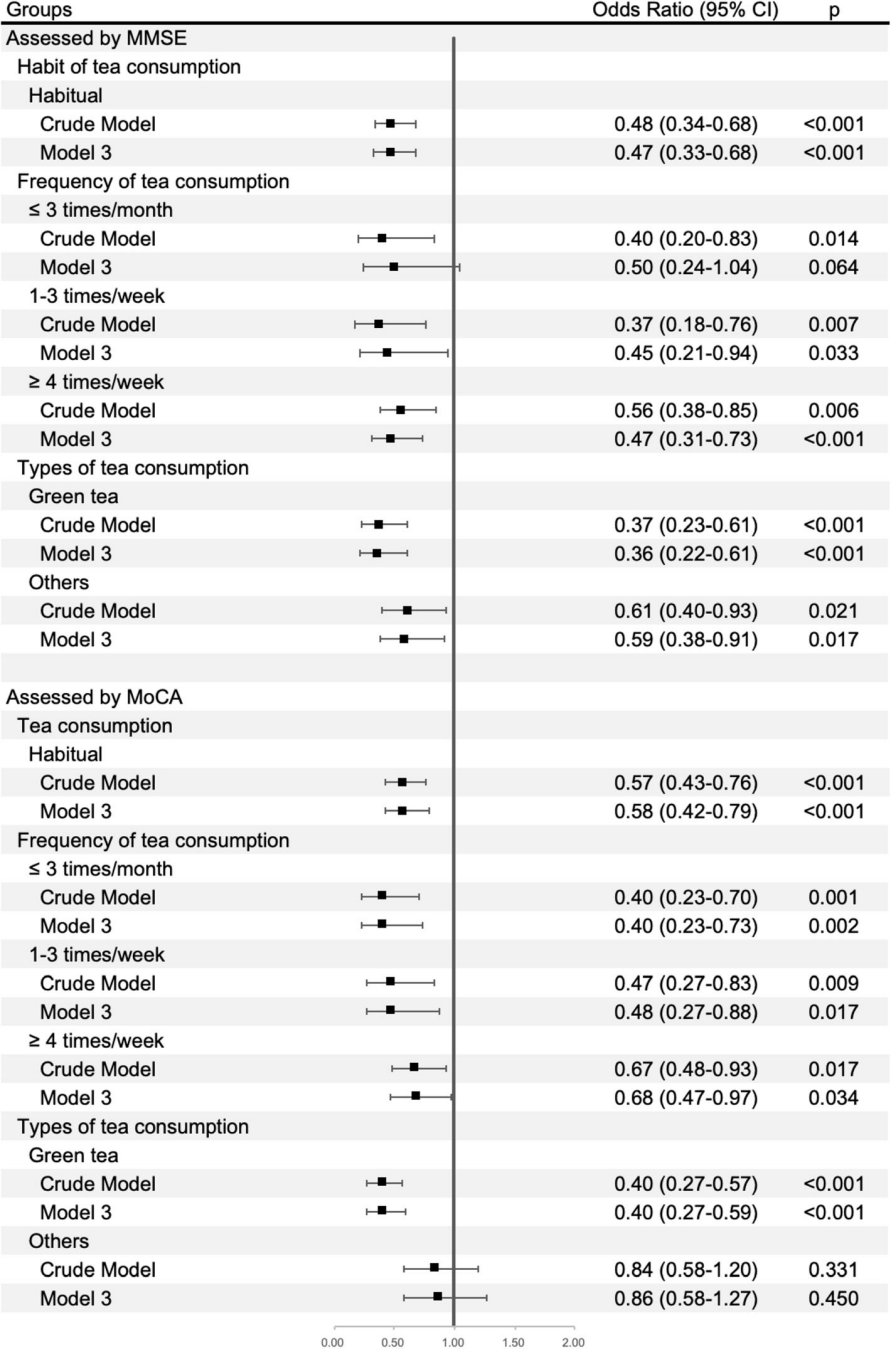
Odds ratios of cognitive impairment stratified by habit, frequency and types of tea consumption. Cognitive function was assessed by MMSE and/or MoCA. We divided participants into two subgroups according cognitive function measurements, and conducted analyses, respectively. In model 3, we adjusted for age, sex, level of education, smoking, alcohol consumption, hypertension, diabetes mellitus, dyslipidemia, plasma concentrations of hs-CRP, BMI, physical activities, and salt intake. The “never” group of tea consumption was treated as the reference

The ORs for cognitive impairment stratified by habit, frequency, and types of tea consumption were given in Fig. 2 and Supplemental Table S1–3. Before adjusting for possible covariates in MMSE subgroup analyses, habitual tea intake was associated with low risks of cognitive impairment (OR 0.48, [95% CI 0.34–0.68]). After adjusting for age, sex, level of education, hs-CRP, smoking, alcohol consumption, hypertension, diabetes mellitus, dyslipidemia, BMI, physical activity, and salt intake (model 3), habitual drinking tea remained associated with a lower likelihood of getting cognitive impairment (OR 0.47, [95% CI 0.33–0.68]). Concerning the frequency of tea consumption, because of the small number of people in the group less than once/month and 1–3 times/month, we combined the two groups into ≤3 times/month for analysis. Tea intake for 1–3 times/week (multivariate OR 0.45, [95% CI 0.21–0.94], model 3) and ≥ 4 times/week (multivariate OR 0.47, [95% CI 0.31–0.73], model 3) showed statistically significant association with reduction of cognitive impairment (p trend < 0.001). The risk of cognitive impairment was lower in green tea consumption than other types. Green tea consumption possessed a significant association with a 64% lower risk of cognitive impairment ([95% CI 0.22–0.61], model 3), while other types showed a 41% lower risk (95% CI 0.38–0.91). In MoCA subgroup analyses, we found similar results. These analyses confirmed the results.

## Discussion

In this large population and community-based study, we investigated the association of habit, frequency, and types of tea consumption with the incidence of cognitive impairment in middle-aged and older adults. We observed that tea consumption was associated with a lower risk of cognitive impairment. Notably, high frequency and green tea consumption significantly decreased the risk of cognitive impairment.

This study reported an inverse association between tea consumption and cognitive impairment and provided evidence for the neuroprotective activity of tea. Our findings were in line with previous studies [12, 13]. Tea contains more tea polyphenols, which might explain the effect of tea on cognitive function. Catechins such as epigallocatechin gallate are the primary polyphenols of green tea. Both in-vivo and in-vitro studies have revealed the neuroprotective action of epigallocatechin gallate [11]. The possible neuroprotective mechanism of tea polyphenols is attributed to anti-oxidative stress, modulation of cell signaling pathways, and metal chelator activity [29–32]. The quantity of catechins is different in different types of tea. Green tea contains more polyphenols compared with black tea or oolong tea [33]. This discrepancy among the diverse types of tea is due to the unique fermentation degrees during the processing. The present study investigated both frequency and types of tea intake and suggested that high-frequency tea consumption and all kinds of types may be protective factors against cognitive decline in middle-aged and older people. Green tea showed a more protective effect than others. This study might provide a basis for exploring treatment to prevent and intervene in cognitive impairment.

Besides, this study focused on cognitive function in the middle-aged. With the development of research on cognitive aging, factors affecting brain structure and function, which may result in cognitive impairment, have continuous and cumulative influences throughout the entire life [34]. Moreover, several studies have reported a subtle cognitive decline in the middle-aged [35]. Therefore, attention must be dedicated to the cognitive function and risk factors in the middle-age. In our study, we investigated adults over the age of 40 and assessed the effect of tea consumption on cognitive decline. Similarly, our findings indicated that habitual tea consumption was associated with the reduced risk of cognitive impairment in both the middle-aged and the elderly. To confirm our results, we also used MoCA to measure general cognitive function and performed the analysis. MoCA with high sensitivity and specificity covers more cognition domains than MMSE [19]. We got similar results. We believed that our findings provide a useful clue for effective prevention and intervention for cognitive disorders at an early stage.

However, several studies did not show the association. Some studies with uncommon tea drinking customs of participants [14, 36], and others with a weak association between tea consumption and cognitive impairment failed to draw any conclusion [37]. As known, cognitive dysfunction was a complex process of various risk factors interactions. Age, sex, education, lifestyle, and other diseases may deteriorate cognitive function [38]. This current study observed the protective effect of tea, especially green tea, on cognitive impairment in the middle-aged and elder.

Limitations of this study need to be addressed. First, this study investigated the association between tea consumption and cognitive function at baseline in the middle-age and the elderly. It cannot draw a direct causal relationship between tea consumption and cognitive impairment. Second, this study utilized MMSE and MoCA to evaluate cognitive function, so we cannot identify diagnosis mild cognitive impairment or dementia. Further longitudinal studies, including daily living function assessments, are needed in order to validate our results. Third, only a few participants completed the MoCA test. We will expand the MoCA group size in future research to confirm the findings. All of these limitations need to be addressed in future researches. 

## Conclusion

In this cross-sectional, community-based, and observational study, we observed that habitual tea consumption, especially high-frequency and green tea consumption, was associated with a lower prevalence of cognitive impairment in middle-aged and older individuals.

## Supplementary Information

**Additional file 1: Supplemental Table S1.** Odds ratio for cognitive impairment stratified by habit of tea consumption. **Supplemental Table S2.** Odds ratio for cognitive impairment stratified by frequency of tea consumption. **Supplemental Table S3.** Odds ratio for cognitive impairment stratified by types of tea consumption.

## Data Availability

The datasets used and analysed during the current study are available from the corresponding author on reasonable request.

## Abbreviations

APAC: Asymptomatic Polyvascular Abnormalities in Community
BMI: Body mass index
hs-CRP: High-sensitivity C-reactive protein
MMSE: Mini-Mental State Examination
MoCA: Montreal Cognitive Assessment
