# Supplementary material for: Association between tea consumption and cognitive impairment in middle-aged and older adults
Source: BMC Geriatr. 2020 Nov 4;20:447. doi: 10.1186/s12877-020-01848-6 (PMC7640442; doi:10.1186/s12877-020-01848-6)
Supplement: Supplementary file 1 — Additional file 1: Supplemental Table S1. Odds ratio for cognitive impairment stratified by habit of tea consumption. Supplemental Table S2. Odds ratio for cognitive impairment stratified by frequency of tea consumption. Supplemental Table S3. Odds ratio for cognitive impairment stratified by types of tea consumption. [file 12877_2020_1848_MOESM1_ESM.docx]

**Supplementary Information**

Supplemental Tables S1-3 are the original data of figure 2.

Supplemental Table S1. Odds ratio for cognitive impairment stratified by habit of tea consumption.

|  | Tea consumption | | |
| --- | --- | --- | --- |
|  | Never | Habitual | |
| Assessed by MMSE |  |  | |
| Case, n (%) |  |  | |
| Cognitive impairment | 150 (76.92) | 45 (23.08) | |
| OR (95% CI) |  |  | |
| Crude model | Reference | 0.48 (0.34-0.68)** | |
| Model 1 | Reference | 0.44 (0.31-0.63)** | |
| Model 2 | Reference | 0.46 (0.32-0.66)** | |
| Model 3 | Reference | 0.47 (0.33-0.68)** | |
| Assessed by MoCA |  |  | |
| Case, n (%) |  | |  |
| Cognitive impairment | 242 (50.95) | | 123 (37.16) |
| OR (95% CI) |  | |  |
| Crude model | Reference | | 0.57 (0.43-0.76)** |
| Model 1 | Reference | | 0.58 (0.43-0.78)** |
| Model 2 | Reference | | 0.60 (0.44-0.80)** |
| Model 3 | Reference | | 0.58 (0.42-0.79)** |

* p <0.05, ** p <0.01. OR, odds ratio; CI, confidence interval.

Model 1: Adjusted for age, sex.

Model 2: Adjusted for age, sex, level of education.

Model 3: Adjusted for age, sex, level of education, smoking, alcohol consumption, hypertension, diabetes mellitus, dyslipidemia, BMI, physical activity, salt intake, and plasma concentrations of hs-CRP.

Supplemental Table S2. Odds ratio for cognitive impairment stratified by frequency of tea consumption.

|  | Frequency of tea consumption | | | | |
| --- | --- | --- | --- | --- | --- |
|  | Never | ≤ 3 times/month | 1-3 times/week | ≥4 times/week | *p* for trend |
| Assessed by MMSE |  |  |  |  |  |
| Case, n (%) |  |  |  |  |  |
| Cognitive impairment | 150 (76.92) | 8 (4.10) | 8 (4.10) | 29 (14.87) |  |
| OR (95% CI) |  |  |  |  |  |
| Crude model | Reference | 0.40 (0.20-0.83)* | 0.37 (0.18-0.76)** | 0.56 (0.38-0.85)** | <0.001 |
| Model 1 | Reference | 0.46 (0.22-0.96)* | 0.44 (0.21-0.91)* | 0.44 (0.29-0.67)** | <0.001 |
| Model 2 | Reference | 0.46 (0.22-0.97)* | 0.46 (0.22-0.97)* | 0.46 (0.30-0.70)** | <0.001 |
| Model 3 | Reference | 0.50 (0.24-1.04) | 0.45 (0.21-0.94)* | 0.47 (0.31-0.73)** | <0.001 |
| Assessed by MoCA |  |  |  |  |  |
| Case, n (%) |  |  |  |  |  |
| Normal | 233 (49.05) | 46 (70.77) | 41 (67.21) | 121 (59.02) |  |
| Cognitive impairment | 242 (50.95) | 19 (29.23) | 20 (32.79) | 84 (40.98) |  |
| OR (95% CI) |  |  |  |  |  |
| Crude model | Reference | 0.40 (0.23-0.70)** | 0.47 (0.27-0.83)** | 0.67(0.48-0.93)* | 0.004 |
| Model 1 | Reference | 0.41 (0.23-0.72)** | 0.48 (0.27-0.86)* | 0.68 (0.48-0.96)* | 0.009 |
| Model 2 | Reference | 0.41 (0.23-0.73)** | 0.52 (0.29-0.92)* | 0.70 (0.49-0.98)* | 0.016 |
| Model 3 | Reference | 0.40 (0.23-0.73)** | 0.48 (0.27-0.88)* | 0.68 (0.47-0.97)* | 0.013 |

* p <0.05, ** p <0.01. OR, odds ratio; CI, confidence interval.

Model 1: Adjusted for age, sex.

Model 2: Adjusted for age, sex, level of education.

Model 3: Adjusted for age, sex, level of education, smoking, alcohol consumption, hypertension, diabetes mellitus, dyslipidemia, BMI, physical activity, salt intake, and plasma concentrations of hs-CRP.

Supplemental Table S3. Odds ratio for cognitive impairment stratified by types of tea consumption.

|  | | Types of tea consumption | | | | |  |
| --- | --- | --- | --- | --- | --- | --- | --- |
|  | | Never | Green tea | | Others | |  |
| Assessed by MMSE | |  |  | |  | |  |
| Case, n (%) | |  |  | |  | |  |
| Cognitive impairment | | 150 (76.92) | 18 (9.23) | | 27 (13.85) | |  |
| OR (95% CI) | |  |  | |  | |  |
| Crude model | | Reference | 0.37 (0.23-0.61)** | | 0.61 (0.40-0.93)* | |  |
| Model 1 | | Reference | 0.33 (0.20-0.55)** | | 0.57 (0.37-0.88)* | |  |
| Model 2 | | Reference | 0.35 (0.21-0.59)** | | 0.58 (0.38-0.90)* | |  |
| Model 3 | | Reference | 0.36 (0.22-0.61)** | | 0.59 (0.38-0.91)* | |  |
| Assessed by MoCA | |  |  | |  | |  |
| Case, n (%) |  | | |  | |  | |
| Cognitive impairment | 242 (50.95) | | | 51 (28.98) | | 72 (46.45) | |
| OR (95% CI) |  | | |  | |  | |
| Crude model | Reference | | | 0.34 (0.27-0.57)** | | 0.84 (0.58-1.20) | |
| Model 1 | Reference | | | 0.40 (0.27-0.58)** | | 0.88 (0.60-1.28) | |
| Model 2 | Reference | | | 0.41 (0.28-0.60)** | | 0.89 (0.61-1.30) | |
| Model 3 | Reference | | | 0.40 (0.27-0.59)** | | 0.86 (0.58-1.27) | |

* p <0.05, ** p <0.01. OR, odds ratio; CI, confidence interval.

Model 1: Adjusted for age, sex.

Model 2: Adjusted for age, sex, level of education.

Model 3: Adjusted for age, sex, level of education, smoking, alcohol consumption, hypertension, diabetes mellitus, dyslipidemia, BMI, physical activity, salt intake, and plasma concentrations of hs-CRP.
